# Supplementary material for: Are carbon emissions trading and green financial instruments synergistic? -Comprehensive quantitative research based on content analysis
Source: PLoS One. 2024 Mar 7;19(3):e0298601. doi: 10.1371/journal.pone.0298601 (PMC10919601; doi:10.1371/journal.pone.0298601)
Supplement: S1 Data — (ZIP) [file pone.0298601.s001.zip › Data/地级市Z_F工作报告.zip!/║╙─╧/┐¬╖Γ╩╨/└┤╫╘╣1⁄2╓┌║┼í╛┬φ┐╦╩2╛▌═°í┐_2009.docx]

各位代表：

现在，我代表市人民政府，向大会报告本届政府过去五年的工作，对今后五年及2009年工作提出建议，请予审议，并请各位政协委员和列席人员提出意见。

一、过去五年工作回顾

2004年以来，本届政府在省委、省政府和市委的正确领导下，深入贯彻落实科学发展观，积极构建和谐社会，团结带领全市人民，抢抓机遇，开拓进取，扎实工作，圆满完成了市十二届人大确定的各项目标任务。

经济实力明显增强。2008年，全市生产总值达到689.4亿元，比上年增长13.1%，高出全省平均增幅1个百分点，是2003年的1.8倍；人均生产总值14713元，是2003年的1.8倍。地方财政一般预算收入26.1亿元，比上年增长28.6%，增幅居全省第2位，是2003年的3倍；其中市本级完成11.1亿元，比上年增长29.3%，是2003年的4.8倍。粮食总产创历史新高，达到49.7亿斤，比上年增长4%。规模以上工业增加值191.4亿元，比上年增长20.9%，增幅居全省第5位，是2003年的2.6倍。第三产业增加值223.3亿元，比上年增长17.5%，增幅居全省第1位，是2003年的2倍。城镇固定资产投资231.6亿元，比上年增长41.2%，增幅居全省第2位，是2003年的5.4倍。社会消费品零售总额258.6亿元，比上年增长23.6%，是2003年的2.3倍。进出口总值18270万美元，比上年增长58%，增幅居全省第5位，是2003年的3倍。经济结构进一步改善，全市三次产业比例由2003年的26∶38∶36调整为22∶46∶32，二、三产业比重较2003年提高了4个百分点。

城乡面貌明显变化。城区道路和城市供水、供电、供热、供气以及排水管网等基础设施进一步完善；旧城改造步伐明显加快；城区内背街小巷基本改造完毕；主次干道无路灯现象基本消除；城市公共交通得到有效改善；城市亮化、绿化、美化水平明显提高。日南、大广高速公路建成通车，高速公路通车里程达到274.7公里。新农村建设取得初步成效，全市农村实现“户户通电”，行政村“村村通油路”。县城规划建设管理水平进一步提高，小城镇建设取得新成绩。

社会事业明显进步。教育事业发展迅速，城乡义务教育阶段学生免除学杂费，农村义务教育阶段学生免费提供教科书。以养老、医疗、失业、工伤、生育等各项社会保险为主要框架的社会保障制度和体系建设全面推进，社会保障能力和水平明显提高。文化、卫生、体育等社会事业全面发展；生态建设、节能减排、和谐社会建设等各项工作都取得了新的成效。

人民生活明显改善。2008年，全市城镇居民人均可支配收入11346元，比上年增长16.1%，是2003年的1.8倍；农民人均纯收入4355元，比上年增长15.7%，是2003年的2.1倍。年末城镇居民储蓄存款余额345.1亿元，是2003年的1.8倍。

2008年，我们遇到的困难比预想的多，取得的成效比预期的好。全市经济增长速度超过全省平均水平，主要经济指标增幅在全省位次普遍大幅前移，呈现出良好的发展态势，标志着我市经济社会进入了跨越式发展的新阶段！

五年来，我们主要做了以下工作：

（一）坚定不移地实施工业强市战略。坚持以工业化为核心，通过培育重点企业、扶持优势产业、发展集群经济、实施项目带动、推进结构调整等措施，不断扩大规模、提高效益。2008年，全市规模以上工业增加值增幅、工业投资增幅、规模以上工业企业实现利润增幅分别跃居全省第5位、第2位和第1位，开封工业发展落后的局面得到了根本扭转。重点企业培育初见成效。全市规模以上工业企业达到968家，比2003年增加562家；主营业务收入超亿元的企业达到193家，比2003年增加180家。24户重点企业2008年完成主营业务收入117.7亿元，其中河南千里机械有限公司等12家重点企业主营业务收入超过5亿元，开封蓝翔车业有限公司主营业务收入超过10亿元。企业上市实现零的突破，开封高压阀门有限公司成为我市首家上市企业。产业支撑作用明显增强。机械设备制造、化工、纺织、食品和医药五大产业完成工业增加值114.6亿元，占全市规模以上工业增加值的59.9%。集群经济加快发展。规划建设了杏花营高新技术产业园区、汪屯精细化工区、边村重工业区，五县工业区建设初具规模。初步形成了以11家海马汽车配件企业为龙头的汽车零部件产业园、以尉氏纺织工业园为龙头的纺织工业基地、以蓝翔车业为龙头的电动车生产基地、以永煤集团开封空分设备有限公司为龙头的空分设备产业集群、以三环木业为龙头的木材加工产业集群。工业投资规模不断加大。实施了开封炭素年产2.2万吨超高功率石墨电极、京源公司2×600MW发电机组、汇源集团果汁加工、晋开公司1830、平煤集团开封东大化工15万吨离子膜烧碱、新大新晶硅电池片切磨专用刃料、永煤集团龙宇化工聚甲醛等236个重点工业项目。五年来，全市累计完成工业投资384.4亿元，是上一个五年的10.3倍。

（二）扎实做好“三农”工作。切实把“三农”工作放在重中之重的位置，认真落实党在农村的各项方针政策，不断促进农村繁荣、农业增效、农民增收。各项支农惠农政策全面落实。2005年提前一年全部免征农业税，实施种粮直补、农资综合直补、良种补贴、农机具购置补贴、家电下乡补贴、生猪补贴、奶牛补贴、退耕还林补贴等制度，落实粮食最低收购价政策，农民由过去每年缴纳各类税费2.6亿元变为现在获得政府补贴5.8亿元。农业生产能力持续增强。累计投资5.3亿元，全面启动三义寨、柳园口及赵口灌区续建配套与节水改造，切实加强农田水利基本建设，新增、改善灌溉除涝面积88.8万亩。实行最严格的耕地保护制度，连续10年实现耕地占补平衡。大力推进国家优质粮食产业工程建设，粮食总产稳定在40亿斤以上。农业结构调整不断深化。以建设优质农产品和畜产品生产加工基地为重点，大力发展优质农产品生产，全市优质农产品种植面积比2003年增加40%；积极发展现代畜牧业，新建标准化养殖小区183个，规模养殖比重由33%提高到48%。加快推进农业产业化经营，农业产业化龙头企业达到64个，各类农民专业合作组织发展到近300个。新农村建设扎实推进。累计投入11.8亿元，新建、改造农村公路4373公里；投入1.5亿元，新建、改造农村中小学校舍29.8万平方米；投入2.2亿元，解决了65.9万农民的饮水安全问题；投入0.8亿元，新建农村户用沼气10.5万户，发展农村便民超市1318个。投入2.1亿元，完成了173个贫困村整村推进建设任务，24万农村人口实现脱贫。完成了800个行政村的村庄规划，切实加强70个示范村、451个整治村基础设施建设，村容村貌明显改观。

（三）大力发展第三产业。充分发挥我市深厚的文化底蕴和丰富的旅游资源优势，以文化旅游业为龙头，以传统服务业为基础，大力发展现代服务业，第三产业呈现出快速发展的良好态势。文体节会活动精彩纷呈。成功举办了第十届亚洲艺术节、首届中国收藏文化（开封）论坛、首届中国陶瓷艺术精品收藏博览会、首届中国（朱仙镇）木版年画艺术节和北京奥运火炬开封传递活动、两届郑开国际马拉松赛、市七运会等一系列大型节会、赛事活动；开封菊花花会被评为改革开放30年“影响中国节庆产业进程30个节庆”之一；我市被中国书法家协会授予“中国书法名城”称号。文化旅游精品不断推出。清明上河园琼林苑建成开放，龙亭湖、包公湖景区美誉度进一步提升；大型水上实景演出《大宋·东京梦华》已成为河南的文化品牌；大型原创歌舞剧《清明上河图》、综艺演出《清明上河·梦幻东京》成为展示宋文化魅力的重要载体，为夜游开封增添了新的内容。文物保护和景区创建工作力度加大。汴绣、开封盘鼓等7个项目入选国家级非物质文化遗产名录；新增朱仙镇清真寺等6处全国重点文物保护单位、河南省博物馆旧址等21处河南省文物保护单位；开展了大运河保护与申遗工作；新增开封府、铁塔公园、中国翰园、焦裕禄纪念园4个国家“4A”级旅游景区，全市国家“4A”级旅游景区达到8个，居全省第2位。文化旅游体制改革积极推进。我市被确定为全省文化改革发展试验区和旅游景区（点）改革试点城市，市歌舞剧院改革试点工作顺利完成；清明上河园上市筹备工作全面启动；开封旅游集团组建准备工作基本就绪。文化旅游基础设施得到改善。改建了市博物馆和大众剧院。景区设施不断完善，开元名都大酒店、中州国际酒店、东京艺术中心等一批高档酒店和文化场馆建成开业，大大提升了我市的旅游接待水平。2008年，全市共接待游客2529.4万人次，实现旅游总收入83.4亿元，分别是2003年的2.6倍和3.3倍。市场建设成效显著。投资近6亿元，建成了东京五金建材港、中大义乌商贸城、千盛百货购物广场、繁塔无公害蔬菜批发市场等大型综合专业市场，升级改造了宋都、梁苑、公园东路等农贸市场。

（四）强力推进改革开放。重点领域和关键环节的改革取得重大突破，开放型经济进入新阶段。企业改革成效显著。坚持以产权制度改革为核心，以妥善安置职工为前提，积极引进战略投资者，51家国有、集体企业通过改革、改组、改制焕发出新的生机和活力。非公有制经济快速发展。坚持放胆、放开、放手、放心，鼓励、支持和引导非公有制经济发展。非公有制经济占全市生产总值的比重达60%，比2003年提高12.8个百分点。事业单位改革扎实推进。市属经营服务型事业单位转企改制工作基本完成，市直教育、卫生系统事业单位人事制度改革取得阶段性成果。行政审批制度改革继续深化。五年共精简行政审批事项160项，在全省率先推行涉企收费“一费制”新模式。其他各项改革取得重大突破。市商业银行成功引进战略投资伙伴，综合指标达到国家四类行标准；农村信用社统一法人改革基本完成。区划调整、乡镇机构改革、农村税费改革等重大改革任务顺利完成。城市管理、粮食流通、财税管理、公路养护、市政公用、水利工程管理等领域改革取得明显成效。对外开放水平进一步提高。健全完善招商引资工作机制和激励机制，着力优化经济发展环境，对外开放迈上新台阶。五年来，全市共引进省外资金397亿元，其中2008年引进省外资金115.2亿元，是2003年的4.4倍。国内500强企业中有11家在我市投资，京源发电、海马汽车配件、龙宇化工聚甲醛等一批投资几十亿元的重大项目落户开封。加强对外交流与合作，先后与韩国永川市、澳大利亚温格卡瑞比郡、杭州市、阳江市缔结为友好城市，与我市缔结友好关系的国内外城市达14个。

（五）加快城市建设步伐。坚持规划先行，建管并重，突出抓好基础设施建设、旧城改造和城市管理等工作。城市基础设施建设不断加强。投资20多亿元，建成了魏都路、内顺城路、金耀路、黄河大街北段、夷山大街北段等60多公里城区道路；对大梁路、中山路、晋安路、向阳路、省府街、寺后街、鼓楼街、铁路北沿街等道路进行了改造整治。完成了东京大道北侧景观林、环城公园一期、二期、三期以及金明大道、郑开大道等道路绿化，城区人均公共绿地面积5.3平方米，比2003年增加1.3平方米。新增供水管网297.2公里、排水管网78公里、燃气管网390.7公里。五县污水处理厂全部建成使用，垃圾处理场正在全面建设。旧城改造强力推进。树立经营城市理念，将城市发展与项目建设有机结合，严格执行城市经营性土地招、拍、挂制度，有效保障城市发展和重点项目建设用地。启动实施了宋都古城风貌保护与重现工程。开封城墙保护一、二、三期工程基本完工，水系一期工程如期完成，水系二期等重点工程扎实推进。五年来，全市共完成危旧房改造80万平方米，建设经济适用住房110.6万平方米。城市管理力度加大。进一步理顺城市管理体制，深入开展创“三城”和窗口行业规范管理优质服务竞赛活动，荣获了省级卫生城市、省园林城市等称号。小城镇建设步伐加快。认真落实省委书记徐光春关于朱仙镇发展的批示精神，稳步推进朱仙镇名镇开发建设。积极发展农村服务业，推进人口、产业及农村生产要素向小城镇集中，全市城镇化率达到37.7%，年均提高2.8个百分点，是我市城镇化推进最快的时期。

（六）积极推进郑汴一体化发展。根据区域对接、资源共享、优势互补、错位发展的总体思路，立足我市产业优势，准确定位产业布局，成立了汴西新区建设指挥部，组建了汴西新区党工委和管委会，切实加强对郑汴一体化和汴西新区建设工作的领导和协调。郑汴对接全面启动。建成了郑开大道，开通了城际公交。推出了两市间通信优惠资费政策，联合发行了“联名世纪一卡通”银行卡，通信同城、金融同城、旅游同城迈出坚实步伐，文化、广电等资源共享稳步推进。规划编制成效显著。编制了汴西新区启动区33平方公里规划、汴西新区中心区8.5平方公里规划、基础设施综合规划、郑汴产业带开封段迁村并居规划和汴西调蓄水库规划及周边城市设计，为汴西新区发展绘就了蓝图。基础设施建设全面推进。累计投资9.7亿元，完成了启动区内9条道路建设，供水、供电、燃气、通讯、绿化、照明等配套工程同时跟进。迁村并居工程已经启动。占地6300亩的汴西城市绿带建设已经展开；汴西调蓄水库列入了河南省重点工程和粮食主产区建设总体规划。产业布局有序展开。郑汴产业带开封段建成投产项目38个，完成投资34.2亿元；在建项目6个，已完成投资7.5亿元；入驻和选址项目11个，计划总投资69.2亿元。汴西新区建设已经成为促进开封经济跨越式发展的强力引擎。

（七）大力发展社会事业。深入实施科教兴汴战略，完成各类科技计划项目825项，申请专利1138件，天民种业超级小麦项目荣获国家科技进步二等奖。认真做好社会保障和就业再就业工作，多渠道帮扶3.5万“4050”人员实现再就业，动态消除“零就业家庭”。累计发放小额担保贷款5.3亿元，贷款额居全省第1位、全国省辖市前列；年输出劳动力110多万人次，比2003年翻了一番。连续五年提高企业退休人员基本养老金标准，由人均432元提高到1028元。城镇职工失业保险和工伤保险覆盖面不断扩大。城市低保标准大幅提高，城市低保对象基本生活得到保障。农村低保制度建立实施，农村五保政策较好落实，新改扩建农村敬老院52所，五保对象集中供养率达到42.1%。巩固提高“两基”成果，“两基”工作顺利通过国家验收。全面完成农村中小学D级危房改造任务，农村办学条件明显改善。加大教育资源优化整合力度，大力推进职业教育，各类学校办学水平进一步提高。公共卫生事业得到加强，市、县疾病预防控制体系建设全部完成，城镇医院和乡镇卫生院基础设施明显改善；全面推行城乡医疗保险，参加新型农村合作医疗的农民达到336.6万人，参加城镇职工、居民和困难企业退休职工医疗保险的人数超过70万人，基本实现了全市社会医疗保险全覆盖。经济适用住房、廉租住房建设步伐加快，住房保障水平显著提高。全民健身运动深入开展，竞技体育水平不断提高。继续稳定低生育水平，人口自然增长率控制在5.3‰以内。切实加强社区建设，社区功能不断完善。认真开展产品质量和食品药品安全专项治理，全市食品、药品抽检合格率分别比2003年提高15个百分点和8.6个百分点。切实加强环境保护，圆满完成节能减排工作目标，化学需氧量、二氧化硫排放量分别比“十五”末削减11.4%和56.8%，城市空气质量优良天数达到336天，大气质量明显改善。

（八）努力构建和谐社会。坚持以人为本，执政为民，累计投入近50亿元，坚持每年都为人民群众办若干件实事、好事，得到了人民群众的称赞和拥护。坚持依法行政，自觉接受人大监督，支持政协参政议政，认真办理人大议案、人大代表建议和政协提案。深入开展普法和依法治市工作。切实加强社会治安综合治理，扎实推进平安创建活动，深入开展严打专项斗争，“警民一家亲”活动成效显著，人民群众安全感明显增强。高度重视人民群众来信来访，着力解决人民群众关心的难点热点问题。全面加强安全生产管理，建立健全应急机制，全市社会大局和安全生产形势总体稳定。广泛开展群众性精神文明创建活动，先后创建省级以上文明单位211个，全民素质和文明程度不断提高。积极推进村民自治，顺利完成村委会、居委会换届选举。双拥工作成效显著，第5次荣获“全国双拥模范城”称号。加强同各民主党派、工商联、无党派人士和人民团体的联系，虚心听取各界人士的意见、建议。认真落实党风廉政建设责任制，切实加大案件查处力度，廉政建设不断加强。积极支援四川地震灾区抗震救灾，扎实做好对口援建工作。驻汴解放军和武警部队在抗震救灾、维护社会稳定、支持开封经济建设等方面做出了重要贡献。民族宗教、社会科学、新闻出版、审计、统计、外事、侨务、对台、档案、史志、人防、气象等工作都取得了新的成绩。

各位代表：

过去的五年是我市经济实力大幅提升、发展后劲显著增强、城乡面貌发生巨大变化、人民群众得到实惠最多的五年。回顾过去五年的工作，我们深刻体会到：这一时期最坚定的发展思路是实施“六大战略”，推进“三个跨越”。我们始终坚持以科学发展观为指导，把发展当作第一要务，唱响奋力实现经济跨越式发展的主旋律，充分运用各种有利条件，坚持一张蓝图绘到底，坚定不移地实施工业强市、开放引进、旅游带动、科教兴汴、城市化和可持续发展战略，加快老工业基地向新型工业化城市跨越、传统农业向现代农业跨越、文化旅游大市向文化旅游强市跨越。这一时期最强劲的发展动力是解放思想、改革开放。我们始终把解放思想作为应对新挑战、开创新局面的一大法宝，努力把中央、省委要求与开封实际紧密结合起来，不断创新发展理念。坚持用改革和发展的办法解决前进中的问题，深入实施开放引进战略，强力推动改革向纵深推进、向广度拓展，广大干部群众的思想观念发生深刻变化，有效调动了各方面的积极性、创造性，进一步解放和发展了社会生产力。这一时期最根本的发展方法是统筹兼顾、协调推进。我们坚持在全面提升二、三产业发展水平的同时，始终重视农业生产，农业基础地位得到巩固和加强，为国家粮食安全做出了重要贡献；在着力推进郑汴一体化发展的同时，加快发展县域经济；在推进国有经济战略性调整的同时，大力发展非公有制经济，形成了全面协调发展的新格局。这一时期最浓厚的发展氛围是人心思进、求真务实。我们牢固树立正确的政绩观，坚持靠发展解决问题，以实干凝聚人心，扎扎实实做了一些打基础、利长远的工作，在全市上下营造思发展、议发展、谋发展的浓厚氛围，努力在创新中跨越，在开放中振兴，在奋斗中崛起。这一时期最可喜的发展成果是民生改善、群众受益。我们坚持把实现好、维护好、发展好人民群众的根本利益作为工作出发点和落脚点，切实从社会最关心的就学、就医、就业、社会保障等难点问题入手，为广大人民群众办了一些实事好事，解决民生问题的投入比以往任何时候都多，改善民生问题的成效比以往任何时候都好。

各位代表：

过去的五年，全市经济社会取得了突破性进展，开创了跨越式发展的良好局面。这些成绩的取得，是省委、省政府和市委正确领导的结果，是各级人大、政协积极支持的结果，是全市人民共同奋斗的结果。值此，我代表市人民政府，向全市人民和各民主党派、工商联、无党派人士以及社会各界，向驻汴人民解放军、武警官兵和民兵预备役部队，向关心、支持开封发展的同志们、朋友们致以崇高的敬意和衷心的感谢！

在肯定成绩的同时，我们也清醒地看到开封发展中存在的突出矛盾和问题：当前，全球性的金融危机对我市影响日益加剧，经济下行风险加大；我市经济总体实力不强，经济结构不优，自主创新能力不强，面临着扩大总量与优化结构的双重压力；城乡发展不平衡，县域经济薄弱，农村基础设施建设滞后、农业生产水平较低、农民增收难的问题依然突出，城乡建设和管理水平有待提高；少数部门大局意识、服务意识不强，经济发展环境仍需改善等。对这些问题，我们将高度重视，认真加以解决。

二、今后五年政府工作的总体要求和奋斗目标

今后五年，是开封经济社会实现新突破、新发展、新跨越的关键时期，我们的发展任务十分繁重，同时也面临着难得的历史机遇。全球金融危机导致国际国内产业转移加快，为我们拓展资源配置空间、承接产业转移提供了更为有利的条件；党的十七大推动经济社会进入了科学发展新时期，中央实施促进中部地区崛起和建设社会主义新农村重大战略，继续加强和改善宏观调控，实施积极的财政政策和适度宽松的货币政策，为我们提供了更为良好的宏观发展环境；省委、省政府实施“两大跨越”，推进中原城市群建设，积极构建“一极两圈三层”城乡统筹发展新格局，全力打造“郑汴新区”核心增长极，重新确立了开封在全省的战略核心地位，为我们提供了新的发展契机；当前，开封正处于工业化、城镇化和农业现代化快速发展的新时期，具备了良好的发展基础。我们必须认清新形势，抢抓新机遇，努力实现科学发展、和谐发展、跨越式发展。

按照市九次党代会提出的指导思想和目标任务，今后五年政府工作的总体要求是：全面贯彻落实科学发展观，以实现经济社会又好又快发展为目标，坚定不移地实施工业强市、开放引进等“六大战略”，围绕加快老工业基地向新型工业化城市跨越、传统农业向现代农业跨越、文化旅游大市向文化旅游强市跨越，着力推进经济结构调整，着力加快汴西新区建设，着力深化改革和扩大开放，着力解决事关民生和社会和谐的重大问题，为建设富裕、民主、文明、和谐的新开封而努力奋斗。

——经济建设实现新跨越。到2013年，全市生产总值比2008年翻一番，力争突破1400亿元；财政一般预算收入力争突破60亿元，其中市本级达到25亿元。农业稳定发展，工业做大做强，服务业全面提升，自主创新能力明显增强，社会主义新农村建设扎实推进，城市竞争力显著提高。

——汴西新区建设实现新突破。坚持高标准规划、高水平建设，努力把汴西新区建成既传承历史文化又具有浓郁时代气息，布局合理、功能完善的现代化、生态型新城区，成为全省经济社会发展的核心增长极、改革开放的试验区、科学发展的示范区。

——文化建设迈出新步伐。文化资源优势得到充分发挥，文化事业加快发展，文化产业占全市经济的比重明显提高。基层公共文化设施进一步完善，公共文化产品服务和供给能力显著提升，文化软实力不断增强。

——生态文明建设取得新成效。能源资源利用效率明显提升，主要污染物排放量有效控制，到2013年单位生产总值能耗比2008年降低20%左右。林业生态市初步建成，环境质量明显改善，经济发展与人口、资源、环境趋于协调。

——和谐开封建设开创新局面。精神文明、民主法制建设进一步加强。城乡居民收入大幅增加，生活水平和质量明显提高，城乡义务教育均衡发展，基本医疗卫生服务明显改善，劳动就业更加充分，覆盖城乡居民的社会保障体系初步建立，绝对贫困现象基本消除。

各位代表：

我们这个有着悠久历史、灿烂文化的千年古都，目前已经进入了一个跨越式发展的新阶段。我们相信，再经过五年的努力，一个更有实力、更具活力、更富魅力的新开封，必将焕发出更加璀璨夺目的光彩！

三、2009年政府主要工作

今年可能是新世纪以来经济发展最为困难的一年，也是蕴含重大机遇的一年。当前国际金融危机尚未见底，对实体经济的影响进一步加深，一些深层次矛盾凸显，使得我市面临的形势更加复杂、更加严峻。面对困难，我们既要增强忧患意识，立足于应对挑战，把困难估计得更充分一些，把措施准备得更周密一些，努力把不利影响降到最低限度；更要增强机遇意识，坚定必胜信心，努力化危为机，借机发展。根据当前严峻的经济形势和今后五年的目标任务，今年我市政府工作的首要任务是保持经济平稳较快发展，保持经济社会跨越式发展良好态势。全市上下必须按照“扩内需、促增长、调结构、抓项目、保态势”的总体思路，努力实现经济社会又好又快跨越式发展。

今年我市经济和社会发展的主要预期目标是：生产总值增长11%左右；地方财政一般预算收入增长11%，其中市本级增长11%；全社会固定资产投资增长25%；社会消费品零售总额增长17%；出口总额增长10%，实际利用外资增长13%；居民消费价格涨幅控制在4%左右；城镇居民人均可支配收入增长7%，农民人均纯收入增长6%；城镇登记失业率控制在4.5%以内；人口自然增长率控制在6.5‰以下；单位生产总值能耗降低5%；化学需氧量排放量下降3.7%，二氧化硫排放量完成“十一五”总量控制任务。重点做好以下工作：

（一）全力保持经济平稳较快发展良好态势，加快推进经济跨越式发展。进一步增强经济运行调节的预见性、科学性，采取更直接、更有力的政策措施，统筹兼顾、突出重点，务求实效。着力扩大投资需求。立足我市基础条件和产业优势，瞄准国家产业政策和投资方向，着力谋划实施一批科技含量高、投资规模大、带动能力强的重大建设项目，争取纳入国家和省的项目投资计划。同时，落实重点项目协调推进机制，力争全市166个重点项目完成投资160亿元。着力扩大消费需求。采取更加积极的措施，提高城乡居民收入，积极开拓消费市场。推进事业单位工资制度改革。完善城乡社会救助体系和优抚保障制度。大力培育消费热点，重点扩大住房消费、农村消费和服务消费。积极发展商业连锁经营，支持电子商务平台建设。继续实施新农村现代流通网络工程和“万村千乡市场工程”，新建400个农村连锁超市，覆盖全市80%以上行政村。实施两个大型农产品批发市场升级改造项目。落实好“家电下乡”补贴工作。着力保持对外贸易稳定增长。落实好国家出口退税政策，加大对特耐集团、兴化精细化工等重点出口创汇企业的扶持力度，鼓励和支持企业开拓国际市场。着力为企业排忧解难。大力开展“企业服务年”活动，主动帮助企业解决生产经营中的资金、环境等问题。继续组织好银企合作，引导金融机构加大信贷投放，多渠道、多形式解决好企业的资金问题。强力推行涉企收费“一费制”和项目代理制，进一步优化发展环境。

（二）强力开发汴西新区，加快推进郑汴新区建设。抓住省委、省政府全力打造“郑汴新区”、建设全省经济社会发展核心增长极的重大机遇，调整充实汴西新区组织机构，加强汴西新区内部工作协调以及与“郑汴新区”的对接，强力推进汴西新区的开发和建设。积极搭建投融资平台。抢抓国家实施适度宽松货币政策的机遇，组建汴西新区基础设施投资公司，加强与各金融机构和投资集团的沟通和衔接，进一步创新工作机制，拓宽投融资渠道，积极为汴西新区建设筹措资金。加快基础设施和项目建设。全面启动汴西新区的道路、水系、管网等基础设施和已审批项目建设。开工建设汴西调蓄水库。认真做好郑汴轻轨、郑徐高速铁路客运站建设前期工作，争取年内开工建设。启动职业教育园区建设。加大对职业教育资源的整合力度，做好职业教育园区规划，全面启动入园学校建设，力争三年内建成总面积5000亩以上、在校生6万人以上的综合性教育园区。扎实推进迁村并居。科学规划，完善方案，周密组织，加快推进阎砦、毛砦迁村并居工程建设，做好启动区内迁村并居各项工作，争取三年内合并建成4个新型社区。科学展开产业布局。切实抓好汽车零部件产业园、空分特色产业园的规划和建设工作，加大招商引资力度，力争再有一批科技含量高、带动能力强的大项目落户汴西新区。同时，加快配套服务设施建设，完善新区功能，提高承载能力，努力把汴西新区建设成为新的产业集聚区和城乡一体化发展的现代复合型城区。

（三）坚定不移地实施工业强市战略，加快推进产业结构优化升级。紧紧围绕工业强市目标，大力推进工业结构优化升级，全面提高工业经济效益。着力培育重点企业。加强对重点企业经济运行的分析监控，及时协调解决发展中遇到的各种困难和问题，争取再有3～5户企业主营业务收入超过10亿元。积极引入战略投资者，支持企业实施战略重组，尽快培育一批大型企业或企业集团。加快平原非标、新大新科技上市步伐。突出发展优势产业和产业集聚区。继续抓好机械设备制造、纺织、食品、化工、医药等优势产业发展，重点培育汽车零部件、空分设备、精细化工、通许电动车、尉氏纺织、杞县新材料和兰考木材加工等产业集聚区。狠抓重点工业项目建设。重点抓好晋开公司百万吨总氨、永煤集团开封大型空分设备制造、龙宇化工聚甲醛、新大新晶硅电池片切磨专用刃料生产等90个工业项目，力争完成投资106.9亿元。加快科技创新步伐。强化科技创新在培育高新技术产业中的集聚辐射和带动作用，增强企业自主创新能力，谋划、催生一批先进制造业、新材料、光电和生物工程等高新技术项目，逐步建立面向未来的战略支撑产业。大力发展非公有制经济。毫不动摇地鼓励、支持、引导非公有制经济发展，加快推进中小企业担保和社会化服务体系建设，扩大中小企业投资担保股份有限公司资本规模，为中小企业提供更加方便快捷的资金服务。完善中小企业发展配套政策，制定开封市民营企业投诉中心办事程序，加快构建市县两级维权服务网络，促进非公有制经济快速发展。

（四）切实加强“三农”工作，加快推进农村改革与发展。围绕稳粮、增收、强基础、重民生，进一步落实惠农政策，深化农村改革，巩固和发展农业、农村好形势。进一步加大“三农”投入力度。坚持并完善农业支持保护和补贴制度，大幅度增加对农村基础设施建设和社会事业发展的投入，大幅度提高政府土地出让收益、耕地占用税新增收入用于农业的比例，大幅度增加农村公益性建设项目的投入。认真抓好国家粮食核心区建设工程。全面启动国家粮食战略工程开封核心区建设，加快大型灌区续建配套与节水改造步伐，力争开工涡河、贾鲁河、惠济河骨干河道治理工程，加强农田水利基本建设，提高抗灾夺丰收能力。稳步推进优质粮食产业工程，加快五县国家大型商品粮基地和沿黄县区优质粳稻生产基地建设，确保全年粮食种植面积达到700万亩，总产在50亿斤左右。着力调整农业结构。大力发展优势农业，努力打造4个百万亩优质农产品产业带。加快农产品标准化生产示范区、无公害农产品生产示范基地建设。积极发展现代畜牧业，着力建设生猪、奶牛、肉牛、家禽四大养殖基地。加大对中原皓月、禾丰肉鸡、大用集团等农业产业化龙头企业的扶持力度，大力发展农民专业合作组织。加强重大动物疫病防控，落实农畜产品市场准入制度，确保农畜产品质量安全。全面推进农村各项改革。稳定和完善农村基本经营制度，加强土地承包经营权流转管理和服务，建立健全土地承包经营权流转市场，按照依法自愿有偿原则，发展多种形式的适度规模经营。改善和创新农村金融服务，加大涉农金融政策支持力度；充分发挥农行、农信社金融支农主力军作用；完善农村信贷担保机制。加快集体林权制度改革。深化农村综合改革，增强乡镇政府社会管理和公共服务能力。扎实推进新农村建设。继续扩大新农村建设示范村和整治村范围，增加并整合支农资金，搞好村镇体系规划，加快农村沼气、饮水、电力、道路等基础设施建设，着力抓好村容村貌整治。加大扶贫开发力度，完成59个贫困村的整村推进扶贫任务。

（五）大力发展文化产业，加快推进文化大发展、大繁荣。紧紧抓住省委、省政府加快推进文化改革发展试验区建设的机遇，充分挖掘开封文化内涵，主打宋文化品牌，加快推进文化体制改革，完善公共文化服务体系，大力发展文化产业，努力实现文化与经济的融合，推动文化发展与繁荣。加快文化改革发展试验区建设。制定完善文化改革发展试验区工作计划、评价体系和考核标准，着手编制文化改革发展试验区建设规划，创建全国知名的文化产业示范园区，争当文化强省排头兵。深化文化体制改革。加快经营性文化事业单位转企改制，积极推进文艺院团改革取得实质性进展，认真做好电影管理职能调整划转工作。加大文化资源整合力度，着力培育文化产业龙头企业，形成以产业集团为骨干、各类文化企业共同发展的文化产业集聚区。着力发展文化产业。全面落实文化产业扶持政策，设立文化产业发展专项基金，重点发展文化旅游、文艺演出、工艺美术、休闲娱乐、传媒出版以及会展、收藏、动漫等文化产业，积极发展特色文化产业集聚区，促进文化产业全方位发展。精心打造文化品牌。着力推进开封城墙修复保护、四河连五湖水系工程、宋宫主题公园、中原艺术品交易市场、中国收藏文化（开封）论坛坛址、清明上河园扩建、开封府扩建、大相国寺北扩、繁塔禹王台大规模整修、动漫城建设、“城摞城”博物馆等精品工程建设；推动开封汴绣、朱仙镇木版年画、北宋官瓷等特色文化产业区块发展；进一步完善《大宋·东京梦华》水上实景演出；继续办好中国收藏文化（开封）论坛，扩大开封文化影响力，建设文化开封。

（六）着力提高服务业发展水平，加快推进第三产业全面升级。按照市场化、产业化、社会化的方向，以增加供给、优化结构、提高质量、扩大就业为目标，加快发展生产性服务业，充实提升生活性服务业，努力形成体系完整、布局合理、特色突出、聚集力强、辐射面广的现代服务业发展新格局。加快发展旅游业。加大旅游资源整合力度，完成开封旅游集团组建工作。加快清明上河园改制上市步伐，着力培育一批旅游龙头企业。深度开发旅游资源，积极开展清明上河园创建“5A”级景区工作，培育更多的旅游品牌。积极引进国内外知名旅游企业、旅游经营机构参与我市旅游业开发，提升旅游行业整体水平，促进餐饮、住宿、购物、交通、娱乐、商务等相关服务业发展，拉长旅游产业链条。提升传统服务业发展水平。大力支持商贸餐饮等传统服务业发展，扶持有条件的“老字号”、“老品牌”做大做强。制定开封名吃发展规划，建立食品工业园区，促进传统名吃实现规模化经营、产业化发展。着力发展现代服务业。积极发展现代物流业，建设开封市农副产品物流园区；改善金融生态环境，吸引国内外金融机构在开封设立分支机构，大力发展金融服务业；着力谋划一批现代服务业项目，推进信息、咨询、中介等成长性强的现代服务业发展。

（七）加强旧城改造和城市建设管理，加快推进城镇化步伐。坚持城市规划与城市建设并重、新城开发与旧城改造并举，全面开展规划区控制性详规编制工作，更好地指导城市建设。强力推进旧城改造。抓住宋都古城风貌保护与重现工程列入省重点项目的机遇，加大基础设施建设力度，完善老城区城市功能。完成水系二期工程建设，同步启动周边地块开发。实施开封城墙保护四期工程，完成大梁门以北西城墙修复及景观建设。在确保安置用房到位的基础上，全面启动老城区1号、2号、3号地块拆迁改造。进一步加强城市管理。继续深化创“三城”活动，着力提高市民文明素质和城市形象；突出抓好市容环境、市政设施、园林绿化等方面的精细化管理，提升城市品位，努力塑造历史文化与现代文明交相辉映的城市风貌，建设中原宜居城市。加快县城和小城镇建设。切实加强县城管理，完善县城基础设施，提高县城规划建设水平。强力推进朱仙镇开发，重塑历史文化名镇风采。着力抓好沿国道、省道小城镇的规划与建设，推进城乡一体化发展。

（八）扩大改革开放，加快推进开放型经济发展。坚持以改革为动力，深入推进体制机制创新，通过改革振奋精神、增强信心、促进发展、保持稳定。扎实推进企业改革和战略重组。鼓励优势企业兼并重组落后企业、困难企业，充分利用有效资产，稳定生产和就业；鼓励强强联合，提高规模效益，实现优势互补；鼓励关联产业、上下游企业联合重组，实现一体化经营，提高抗风险能力。加强与世界和国内500强大型企业的战略合作，进一步提升我市企业的知名度和竞争力。深化财税、价格体制等各项改革。进一步完善财政预算体系。按照国家、省统一部署，实施好新修订的增值税、营业税、消费税暂行条例及燃油税费改革方案。从财政状况较好的县入手，逐步推进趸售电价体制改革。同时，深化医药卫生体制、金融体制改革，积极推进事业单位人事制度改革，继续推进科技、教育、社会管理等领域改革。深入实施开放引进战略。紧紧抓住当前世界金融危机蕴含的机遇，积极主动承接沿海发达地区产业转移，以更大的开放规模、更高的开放水平推动开放型经济发展。围绕培育壮大支柱产业、产业集聚区和重点企业，精心搞好项目策划，大力开展项目招商，加强产业对接，争取促成、建设一批有利于增强我市产业发展后劲的重大项目，推动产业结构优化升级。充分发挥企业在招商引资中的主体作用，积极引进战略合作伙伴，促进企业做大做强。大力开展对外劳务合作，抓好外派劳务，鼓励和支持有条件的企业到境外承包工程。办好中国开封第27届菊花花会，筹备好全国第10届菊花展览会。

（九）强化节能减排，加快推进生态文明建设。把加强资源节约和环境保护作为转变发展方式的重要抓手，完善节能减排指标体系、监测体系和考核体系，努力实现节约发展、清洁发展、可持续发展。强力推进节能降耗。突出抓好工业、交通、建筑等关键领域节能工作，加快淘汰行业落后产能。严把新建项目准入关，严格“两高一资”项目审查。加强电力、有色金属、建材、化工等重点行业节能降耗，推进余热余压利用、能量系统优化等重点节能工程建设。积极推广绿色照明工程。认真落实《公共机构节能条例》，推进公共机构节能工作。加强环境保护和生态建设。集中环保专项资金，重点支持市、县环境监控系统平台建设，完成全市城市污水处理厂COD和氨氮自动监控设备更新改造。已建成污水处理厂全面稳定达标运营。推进城市生活垃圾无害化处理项目建设。加强对惠济河、贾鲁河、涡河等重点流域环境综合整治。对造纸、制药等行业18家企业实施废水限期治理。认真实施林业生态市建设规划，完成营造林面积22万亩。建立健全节约资源、保护环境长效机制。坚持节约集约用地制度，提高资源利用效率。着力建设循环经济城市，大力发展节能环保产业。完善排污收费机制。鼓励企业加大环保投入，完善环保设施，严格执行环境法规和排放标准。深入开展全民节能减排行动，倡导节约型生产方式、消费模式和生活习惯。

（十）致力改善民生，加快推进各项社会事业发展。切实解决涉及群众切身利益的增收、就业、就学、就医、社会保障、住房保障和食品安全等问题，促进社会和谐稳定。千方百计扩大就业。实施更加积极的就业政策，多渠道开发就业岗位，年内城镇新增就业6万人，实现下岗失业人员再就业2.5万人。进一步开发公益性就业岗位，完善公共就业服务和就业援助制度，动态解决就业困难群众和“零就业家庭”人员就业问题。全面推动以创业促进就业，落实好农民工返乡创业政策，鼓励高校毕业生、失业人员等不同群体积极创业。继续实施“农村劳动力技能就业计划”、“阳光工程”和“雨露计划”，提高就业能力，缓解就业压力。提高社会保障水平。大力开展“社会保障年”活动，力争将未参加社会保险的人员全部纳入覆盖范围。多渠道筹措社会保障基金，逐步建立社会保险、社会救助、社会福利、慈善事业相衔接的覆盖城乡居民的社会保障体系。继续完善城镇职工基本养老保险制度。全面推进社会基本医疗保险工作，提高参保率，农民平均参合率达到95%，参合标准提高到100元。加大城乡医疗救助力度。基本解决困难企业退休职工医疗保险问题。加大保障性住房建设力度。开工建设经济适用住房50万平方米、竣工40万平方米，为城镇低收入家庭提供4000套以上经济适用住房。做好城市低收入家庭廉租住房保障工作，对9000户低收入住房困难户发放租赁补贴，对申请廉租住房保障并符合政策条件的低保家庭做到应保尽保；新建、回购廉租住房400套，对低保住房困难家庭逐步采取实物配租方式解决住房困难。着力帮扶特殊困难群体。进一步完善城乡低保制度，提高城乡低保标准。巩固农村五保对象集中供养率，提高供养标准。积极发展社会慈善事业、养老事业和残疾人事业，加快“蓝天计划”市儿童福利院和市流浪未成年人保护中心建设。继续抓好对口援建工作，确保按期完成援建任务。优先发展教育事业。提高城乡义务教育阶段学校生均公用经费标准，落实好农村义务教育“两免一补”和免除城市义务教育阶段学生学杂费政策。认真解决农民工子女在流入地免费接受义务教育问题。积极化解农村“普九”债务，力争3年内基本解决。加大高中教育阶段教育投入。积极实施职教攻坚计划，整合职教资源，加快开封大学新校区和新高职学院建设。发展农村职业教育并逐步免费。建立市校合作机制，全力支持河南大学搞好省部共建。支持在汴高等院校、中等专业学校加快发展。健全医疗卫生服务体系。加强公共卫生体系和农村卫生三级网络建设，大力发展城市社区卫生服务，完善新型农村合作医疗制度。扩大农村免费公共卫生服务和免费防疫范围，增加重大传染病、慢性病和职业病防治专项投入，继续实施免费为贫困白内障患者复明手术。市中医院病房楼年底前主体工程竣工。落实好计划生育基本国策。完善计划生育利益导向机制，稳定低生育水平，提高出生人口质量。加强公共文化服务体系建设。大力建设文化惠民工程，实施好乡镇综合文化站、行政村农民体育健身工程、农家书屋等项目。继续实施“千场舞台艺术送农民”和农村数字电影放映工程。广泛开展全民健身运动，继续办好郑开国际马拉松赛。强化安全生产和食品药品安全监管工作。牢固树立安全发展理念，全面落实安全生产责任制。深入开展安全生产隐患排查治理工作，坚决遏制重特大安全事故发生。积极创建食品安全市，开展整顿和规范市场秩序专项行动以及“质量和安全年”活动，深入开展食品药品安全专项整治，严肃查处产品质量安全违法违规案件。维护社会和谐稳定。加强信访工作，及时合理解决群众反映的问题。加强矛盾纠纷排查调处工作，认真落实信访稳定评估制度，有效防范企业破产、征地拆迁、农村土地纠纷、涉法涉诉等可能引发的群体性事件。积极开展双拥共建活动，巩固和发展军政军民团结。深入开展平安创建活动，加强社会治安综合治理，依法严厉打击各类犯罪。加强青少年思想道德建设，优化青少年成长环境。加强基层政权和民主政治建设，完成社区居委会换届选举。支持工会、共青团、妇联等人民团体充分发挥密切联系群众的桥梁纽带作用和参与社会事务管理作用。继续做好外事、侨务、对台、统计、档案、史志、地震、气象、人防、民族宗教、社会科学、新闻出版等工作。多方筹措资金，再办15件为民实事。今年，我们要继续办好关系人民群众切身利益的实事、好事，努力在改善民生上取得更大成效。

四、转变政府职能，加快推进政府自身建设

今年经济形势异常严峻，各项工作任务异常繁重。我们要积极开展深入学习实践科学发展观活动，扎实推进“两转两提”，切实提高政府公信力、执行力，切实保障经济社会又好又快跨越式发展，以优异成绩迎接新中国成立60周年。

（一）更加注重职能转变和管理创新，努力建设服务型政府。继续转变政府职能，加强制度建设，健全行政决策、执行、咨询、反馈、监督等方面的程序和制度。深化行政管理体制改革，积极稳妥推进政府机构改革，争取上半年完成市政府机构改革，年底前完成县级政府机构改革。围绕服务型政府建设，扎实推进“两转两提”，增强服务意识，提高办事效率和服务水平。

（二）更加注重依法行政和法制建设，努力建设法治政府。严格执行人大及其常委会的决议、决定，自觉接受人大及其常委会的法律监督和工作监督。自觉接受人民政协的民主监督。广泛听取各民主党派、工商联和无党派人士的意见和建议。支持司法、监察、审计等部门依法独立履行职责。重视发挥新闻舆论、网络媒体的监督作用。认真贯彻政府信息公开条例，保障群众知情权，接受群众监督。大力推广电子政务。加强和改进行政服务中心工作。不断优化政府办事规程和行为准则，规范政府审批和行政许可行为。强化行政执法责任制，推进文明执法。健全以行政首长为重点的行政问责制度，加大责任追究力度。

（三）更加注重廉政建设和反腐败工作，努力建设廉洁政府。加强党风廉政建设，着力完善惩治和预防腐败体系，落实党风廉政建设责任制，认真执行领导干部廉洁自律规定，加强领导干部经济责任审计，强化权力运行制约和监督。牢固树立过紧日子的观念，严格控制公务购车用车、会议经费、公务接待费用以及党政机关出国出境经费等支出，坚决制止铺张浪费，做到令行禁止、违者必究。

（四）更加注重作风转变和能力提升，努力建设效能政府。坚持发扬求真务实精神和艰苦奋斗精神，多干打基础、利长远的事，多抓加强基层基础建设的事，努力做出经得起实践、人民、历史检验的实绩。坚持深入实际、深入基层、深入群众，开展调查研究，做到问政于民、问需于民、问计于民，科学决策，少走弯路，使发展思路、工作部署、政策措施更加符合实际、更加符合人民群众愿望。切实改进会风、文风，大力精简会议、文件，坚决反对虚报浮夸、弄虚作假，坚决反对官僚主义、形式主义。以正确政绩观落实科学发展观，更加紧迫地学习新知识、增长新本领，着力把握发展规律，创新发展理念，切实提高为基层、为群众服务的能力。

各位代表！

新的一年，挑战与机遇同在！让我们紧密团结在以胡锦涛同志为总书记的党中央周围，高举中国特色社会主义伟大旗帜，深入贯彻落实科学发展观，在省委、省政府和市委的坚强领导下，团结拼搏，求真务实，开拓进取，保持经济平稳较快发展，保持经济社会跨越式发展良好态势，为夺取全面建设小康社会新胜利，谱写人民美好生活新篇章而努力奋斗！
